# Weight loss and metabolic health effects from energy-restricted Mediterranean and Central-European diets in postmenopausal women: A randomized controlled trial

**DOI:** 10.1038/s41598-018-29495-3

**Published:** 2018-07-24

**Authors:** Joanna Bajerska, Agata Chmurzynska, Agata Muzsik, Patrycja Krzyżanowska, Edyta Mądry, Anna M Malinowska, Jarosław Walkowiak

**Affiliations:** 1 Institute of Human Nutrition and Dietetics, Poznan University of Life Sciences, Wojska Polskiego 31, 60–624 Poznan, Poland; 2 First Subdepartment of Pediatrics, Department of Pediatric Gastroenterology and Metabolism, Poznan University of Medical Sciences, Szpitalna 27/33, 60-572 Poznan, Poland

**Keywords:** Obesity, Outcomes research

## Abstract

We conducted a randomized controlled trial to examine the effect of two energy-restricted diets on body weight (BW), visceral fat (VF) loss, and the risk factors for metabolic syndrome. A total of 144 centrally obese postmenopausal women were assigned to the moderate in fat Mediterranean diet (MED) or to the Central European diet (CED), which is moderate in carbohydrates and high in dietary fiber (DF), for 16 weeks. BW, waist circumference and VF were significantly reduced by 8.8%, 7.0%, and 24.6%, respectively, over the trial (*P* < 0.001), with no difference between groups. A similar trend was seen for total cholesterol, triglycerides, glucose, and blood pressure. Within each diet group, the more adherent participants lost significantly more BW than did their less adherent counterparts. VF was significantly reduced only in women who were more adherent to the CED, and the reduction in VF correlated with an increase in the proportion of DF. Short-term dietary treatment with the CED or the MED was associated with similar improvements in some anthropometric, lipid, and nonlipid parameters; however, adequate adherence to the prescribed diet is important in weight loss success and in achieving improvements in metabolic health.

## Introduction

Menopause is a critical period in a woman’s life, during which body weight gain and redistribution of adipose tissue towards a more central/android phenotype has been observed^1^. There is increasing evidence that visceral obesity is linked to metabolic risk factors of cardiovascular diseases and diabetes^1,2^. Successful strategies for reducing overweight and improving metabolism in postmenopausal women are therefore of the utmost importance^3^.

One dietary model for reducing the incidence of cardiovascular events, which is recommended by the US National Cholesterol Education Program (NCEP)^4^ and the American Heart Association (AHA)^5^, is a low-calorie, low-fat diet. Another dietary model, the so-called Mediterranean diet (MED) is characterized by a relatively high fat intake (up to 40% of total daily calories), of which monounsaturated fatty acids (MUFAs) give 15–25% of the energy^6,7^. The benefits of the MED for body weight loss and for reducing the incidence of cardiovascular events have been pointed out in many studies, where both energy-restricted and *ad libitum* approaches have been applied^8–12^. However, Papadaki & Scott have stated that adoption of the MED by other than Southern Europe populations is difficult, on account of its high cost and the limited availability of these unfamiliar food items, as well as cultural differences that affect food choice^13^. At the same time, interest has increased in diets based on local healthy food items in the prevention or treatment of nutritionally dependent diseases. Eating a traditional balanced Nordic diet has been associated with lower death rates in a Danish cohort study^14^. Similarly to the Nordic region, in the central part of Europe (such as Poland), there are several common food items—including fish (herring), whole grain rye (eaten as rye bread), oats (eaten as oatmeal), barley (eaten as a cereal grain), root vegetables (beetroot), cabbages, berries, apples, and plums—which have much better nutrient profiles than the typical Western diet. The aim of this study was thus to compare the effectiveness of two energy-restricted diets differing in macronutrients on weight loss and improvement in selected risk factors for metabolic syndrome (MetS). The diets used were the MED, which is moderate in fat and has a high proportion of MUFAs, and the so-called Central European diet (CED), which is low in fat, moderate in carbohydrates, and high in dietary fiber (DF) derived from central European food items. Since the outcomes of dietary interventions may depend greatly on adherence to the diet, we considered how this factor contributed to the effects of the CED or the MED.

## Methods

### Trial design and study population

The study involved a 16-week, two-arm parallel group, randomized controlled trial conducted at Poznan University of Life Sciences and at Poznan University of Medical Sciences. This report is a part of a greater study financed by the National Science Centre. The research was approved by the local ethic committee at Poznan University of Medical Sciences (number 603/14) in line with the World Medical Association’s Declaration of Helsinki. The trial registration number was DRKS00012958 (https://www.drks.de/drks_web/) and the date of registration was 6 February 2018. The study protocol, risks, and benefits were explained to each participant. All participants provided their written informed consent to participate in the research study. The study methods and reporting were conducted in accordance with the CONSORT 2010 guidelines. Nonsmoking, postmenopausal women (with absence of menses of over 12 months or serum follicle-stimulating hormone >30 IU/mL) with central obesity (waist circumference; WC ≥ 80 cm), plus at least one other criterion of MetS^15^, who wished to lose weight, were recruited in 2014 through advertisements. Women with type 2 diabetes; monogenic dyslipidemia; a history of cardiovascular disease; use of hypoglycemic, hypolipidemic, anti-inflammatory, or weight loss agents, as well as any drug known to influence liver function; with endocrine disorders or on hormonal replacement therapy were not eligible. The exclusion criteria also included significant weight change in the six months prior to the current study, intolerance or food allergy to key components of the intervention diets, and excessive alcohol consumption (>2 drinks/day).

### Randomization and blinding

The potential participants (n = 269) were screened in an interview to determine eligibility to the study. One hundred and twenty-three women did not meet the inclusion criteria, and two withdrew before being randomized, leaving 144 women to be randomly assigned (1:1) to the MED or the CED. A computer program was used to generate the block randomization sequence (block size 4), using body mass index as the stratification factor. Randomization was performed by study staff who had not been involved in selection of the participants. Participants were blinded to all laboratory data. All study personnel (except the dieticians) were also blinded to the dietary allocation of the participants.

### Study settings

All the planned analyses and dietary interventions were conducted and managed by staff from Poznan University of Life Sciences and from Poznan University of Medical Sciences.

### Dietary intervention

The two supervised dietary intervention arms induced a caloric deficit of ~2.93MJ/day, based on individual energy requirements calculated from indirect calorimetry and physical activity (PA) adjustment. The MED group followed a food plan designed on the basis of the Mediterranean dietary recommendations released in 2010 by the Mediterranean Diet Foundation^6^. To build this menu, typical Mediterranean food products were used. This MED provided approximately 37% energy from total fat, 20% from MUFAs, 9% from polyunsaturated fatty acids (PUFAs), 8% from saturated fatty acids (SFAs), 18% from protein, and 45% energy from carbohydrates. Olive oil was used in every meal and five to seven nuts were served once a day. The CED was based on the recommendations of the NCEP^4^ and the AHA^5^, and was designed to provide 27% energy from total fat, 10% from MUFAs, 9% from PUFAs, 8% from SFAs, 18% from protein, and 55% energy from carbohydrate, with a special emphasis on high levels of DF derived from food items typical of the central European region: cereals (oatmeal and barley), pulses (peas and beans), vegetables (root vegetables, cruciferous vegetables), and fruits (apples, plums). The proportion of soluble to insoluble DF in the CED was 35% to 65%; in the MED this was 20% to 80%. Added salt and refined fats, as well as sugar, were excluded from both diets. Fourteen-day cyclic dietary plans were formulated for both diets using Dietetyk dietary analysis software (Jumar, Poznań, Poland). During the entire intervention period, study participants picked up packaged main meals (covering ~35% daily energy requirements) prepared according to dietician’s recipes by a catering company (Passion Łukasz Bąkowski, Poznań, Poland). Others meals were prepared by the study participants themselves, according to the prescribed dietary plan, including recipes and written instructions to facilitate preparation of meals at home. The participants recorded any foods that were consumed in addition to the study meals, and any foods from the study meals that were not consumed, on a daily compliance questionnaire that was monitored by study staff. Throughout the intervention, volunteers were advised to maintain their usual level of PA and keep other lifestyle factors unchanged.

### Measurements

At baseline (week 0), and at 4, 8, 12, and 16 weeks, participants attended a control appointment for outcome assessment, where measurements of body weight, waist  circumference (WC), blood pressure, PA, and dietary assessment were made. At baseline and at the end of study, all biochemical parameters, dual energy X-ray absorptiometry (DXA) scan, resting metabolic rate (RMR), and nonprotein respiratory quotient (npRQ) analysis were measured.

The primary outcome measures were body weight (BW), visceral fat (VF), and parameters relating to metabolic syndrome risk factors (WC, systolic blood pressure; SBP, diastolic blood pressure; DBP, fasting glucose; GLU, high-density lipoprotein cholesterol; HDL-C, triglyceride; TG). Adherence to the prescribed diets was also determined as one of the primary outcome. The secondary outcomes included fat mass (FM), fat-free mass (FFM), insulin (INS), homeostatic model assessment of insulin resistance (HOMA2-IR), homocysteine (Hcy), total cholesterol (T-C), low-density lipoprotein cholesterol (LDL-C), RMR, npRQ, hunger ratings, and all dietary data.

### Measure of adherence

Dietary adherence was calculated at each time of the four postrandomization points using a Mahalanobis distance equation. This equation was used to measure the difference between the reported and the recommended distribution of energy intake from carbohydrate, fat, MUFAs, and protein. A lower score reflects better adherence and a higher score reflects poorer adherence to the prescribed diets. Details of adherence measuring have been published elsewhere^16^.

### Anthropometric and blood pressure measurements

Height was measured to the nearest 0.1 cm (WPT 200.OC). BW was measured to the nearest 0.1 kg with subjects in bathing suits after an overnight fast using the calibrated scale included in the Bod Pod apparatus. WC was measured at the midpoint between the lowest rib and the top of the iliac crest using nonelastic tape. This measurement was performed twice by a single evaluator. Body composition was assessed by DXA. SBP and DBP were measured using a sphygmomanometer following the standardized criteria of the World Health Organization and the International Society of Hypertension^17^.

### Biochemical parameters

T-C, HDL-C, GLU, and TG concentrations were determined in human serum using a Beckman Coulter AU analyzer. LDL-C concentrations were calculated using the Friedewald formula^18^. Serum INS was determined with the chemiluminescence immunoassay method. The HOMA2-IR index was calculated using the HOMA Calculator v2.2.3 application^19^. Hcy concentrations were measured in serum samples using high performance liquid chromatography (HPLC)^20^.

### Dietary assessment

Dietary intake at baseline and during each control visit (4, 8, 12, and 16) was assessed using a three-day food diary in which the participants were clearly instructed to record information on nonconsecutive days (two weekdays and one weekend day) regarding their food and beverage intake, using household measures. Participants provided details of food product brand names, food preparation methods, and any recipes used. The food and beverage quantities obtained from the food diaries were converted into grams and milliliters and computed using the Dietetyk software. The values for total DF (soluble and insoluble) in the experimental diets were derived from a DF database of common consumption food items^21,22^.

### Self-reported hunger

The level of hunger was compiled at each of the four post-randomization points using visual analog scales (VAS). Participants reported their level of hunger by marking a point on a 100 mm scale with end descriptors ranging from “Not at all” to “Extremely”^23^.

### Resting metabolic rate and respiratory quotient analysis

RMR and npRQ were measured after a twelve-hour fast by indirect calorimetry for 30 minutes (10 minutes of acclimatization and 20 minutes of measurements) while the participants were fasting. Concentrations of CO_2_ and O_2_ were measured using the ventilated hood technique using a Quark RMR (Cosmed, Rome, Italy).

### Physical activity

PA was assessed with the short form of the International Physical Activity Questionnaire (IPAQ-SF). This measure was selected to assess the degree to which participants conformed to the requirement that they not alter their exercise habits during the study, rather than to precisely estimate energy expenditure. The PA results were expressed as metabolic equivalent (MET) minutes per week and classed as inactive (<600 MET/min/week), moderately active (from 600 to 1499 MET/min/week), or active (≥1500 MET/min/week).

### Statistical analysis

Power calculations (α = 0.05, power = 0.8) were performed based on the expected clinically meaningful between-group differences with a weight loss of 4.3 kg, equivalent to a 5% weight loss for an 86 kg person. The targeted final sample size per group was determined using at least 70 participants (G*Power software version 3.1.9.2, Universitat Kiel, Germany). The data were processed using SAS version 9.3 (SAS Institute, Cary, NC, USA) as well as Statistica version 13 for Windows (StatSoft, Tulsa, Oklahoma, USA). Data were analyzed according to an intention-to-treat (ITT) approach, including all participants in each group. Variables were checked for normal distribution; nonnormal variables were log-transformed prior to analysis. For ease of interpretation, arithmetic means and back-transformed variables are presented. Data are expressed as means at the 95% confidence interval, unless otherwise stated. We employed a linear mixed effects model, which has been shown to be a reliable method of handling missing data^24^ so as to compare changes in anthropometrical, biochemical and metabolic parameters, as well as adherence, hunger ratings, and dietary intake between both groups from baseline to 16 weeks across all measurement points. The type of diet was considered a fixed effect and repeated measurements within the condition were considered random effects. The matrix of variances and covariances of the random effects was estimated by the maximum likelihood method using the SAS 9.3 PROC MIXED procedure. The models for the anthropometric, biochemical, and metabolic parameters were adjusted for age. Statistical testing of the differences between the more and less adherent individuals for each diet group was conducted using *t*-tests. All analyses were performed separately within each diet group. The chi-square independence test was used for categorical variables. Pearson correlation was used to calculate the correlation coefficient.

## Results

### Sample characteristics

**Figure 1:**
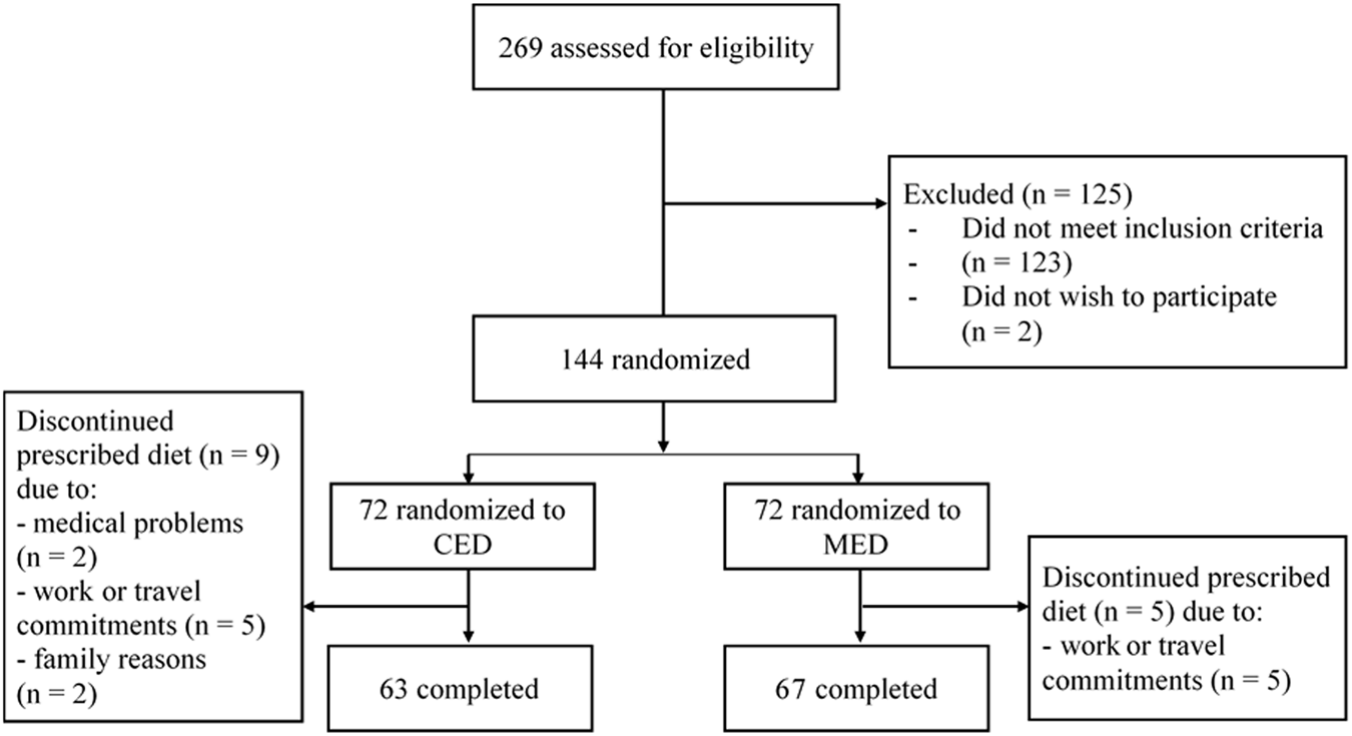
Flow diagram of subjects’ participation in the trial.

An overview of the trial is given in Fig. 1. Fourteen participants (9.7%)—five on the MED and nine on the CED—dropped out of the intervention. One-hundred and thirty women completed the study. There was no instance of participant withdrawal due to being allocated to the CED or the MED group. Upon entry to the study, the postmenopausal women were on average 60.5 year old. Their average age at menopause was 51.0 years (0.5). The whole group was randomized to the CED or the MED and, at baseline, both groups were similar for all variables (Table 1). All participants had at least one MetS criterion in addition to central obesity, which characterized them as a population with or “at risk” of MetS.

**Table 1:** Subject characteristics before and changes in anthropometric parameters after the dietary intervention, analyzed with ITT^a^.

Variables	All	CED group	MED group	*P*-values^b^		
				T	G	T × G
BW (kg)				<0.0001	0.399	0.540
*Baseline*	86.1 (83.9; 88.3)	85.3 (82.3; 88.2)	87.0 (83.6; 90.3)			
*Changes*	−7.6 (−8.2; −7.0)	−7.5 (−8.5; −6.5)	−7.7 (−8.5; −6.9)			
WC (cm)				0.035	0.634	0.970
*Baseline*	105.2 (103.6; 106.8)	105.4 (103.3; 107.4)	105.0 (102.6; 107.5)			
*Changes*	−7.4 (−8.1; −6.8)	−7.5 (−8.5; −6.5)	−7.4 (−8.2; −6.5)			
FM (kg)				<0.0001	0.533	0.747
*Baseline*	39.9 (38.5; 41.3)	39.5 (37.7; 41.3)	40.4 (38.2; 42.6)			
*Changes*	−6.6 (−7.1; −6.1)	−6.7 (−7.4; −6.0)	−6.6 (−7.2; −5.9)			
FFM (kg)				<0.0001	0.456	0.408
*Baseline*	46.2 (45.2; 47.2)	45.8 (44.4; 47.1)	46.6 (45.1; 48.1)			
*Changes*	−0.96 (−1.3; −0.7)	−0.8 (−1.2; −0.4)	−1.1 (−1.6; −0.7)			
VF (kg)				<0.0001	0.810	0.662
*Baseline*	1.06 (1.01; 1.11)	1.06 (1.0; 1.1)	1.07 (1.0; 1.1)			
*Changes*	−0.26 (−0.28; −0.23)	−0.26 (−0.29; −0.23)	−0.25 (−0.28; −0.21)			

Abbreviations: BW: body weight; WC: waist circumference; FM: fat mass; FFM: fat-free mass; VF: visceral fat; T: time; G: group; T × G: time–group interaction; ^a^Values are expressed as means (95% CI); ^b^Linear mixed model for values at each time point, significant at *P* < 0.05; CED: baseline: n = 72; week 4: n = 70; week 8: 68; week 12: n = 65; week 16: n = 63; MED: baseline: n = 72; week 4: n = 72; week 8: 72; week 12: n = 70; week 16: n = 67; in the table values at baseline and changes from baseline to follow-up are presented.

### Changes in anthropometric, lipid, and nonlipid parameters

Both groups had lost weight at 16 weeks (CED: −7.5 kg (95% CI: −8.5, −6.5); MED: −7.7 kg (95% CI: −8.5, −6.9), (*P* < 0.001, time effect), with no difference in weight loss between the groups (*P* = 0.540, interaction effect; Table 1). WC, FM, and VF, as well as GLU, INS, HOMA2-IR, T-C, TG, and blood pressure decreased significantly over the course of the trial, with no difference between groups (Tables 1 and 2). At baseline, there were no significant differences between groups in terms of energy or macronutrient intake (Table 3). At all time points, the reported energy intakes were significantly lower than at baseline (*P* < 0.001, time effect), with no difference between the groups (*P* = 0.968, interaction effect; Table 3). Because of differences in energy intake before and during the dietary intervention, we present macronutrient intakes here as percentage of energy intake. DF was standardized as grams per 4.2MJ. Compared to the MED, the CED group reported higher percentage energy intake from carbohydrate and DF, and lower percentage energy intake from total fat and MUFAs (*P* < 0.0001, interaction effect; Table 3). At all time points, the reported percentage energy intakes from SFAs were significantly lower (*P* < 0.001 time effect) than at baseline, while the percentage energy intake from PUFAs was higher (*P* < 0.001, time effect), with no difference between groups (*P* = 0.322; *P* = 0.943, interaction effect for SFAs and PUFAs respectively, Table 3).

**Table 2:** Subject characteristics before intervention and changes in biochemical and medical parameters after intervention, analyzed with ITT^a^.

Variables	All	CED group	MED group	*P*-values^b^		
				T	G	T × G
GLU (mg/dl)				<0.0001	0.957	0.562
*Baseline*	98.0 (95.9; 100.2)	98.1 (95.2; 101.0)	98.0 (94.8; 101.2)			
*Changes*	−5.9 (−7.3; −4.4)	−5.4 (−7.5; −3.3)	−6.4 (−8.4; −4.3)			
INS (µU/ml)				<0.0001	0.679	0.712
*Baseline*	11.2 (10.2; 12)	11.2 (9.9; 12.6)	11.0 (9.9; 12.0)			
*Changes*	3.3 (−3.9; −2.7)	−3.1 (−4.0; −2.3)	−3.5 (−4.3; −2.7)			
HOMA2-IR				<0.0001	0.649	0.689
*Baseline*	1.46 (1.35; 1.57)	1.48 (1.3; 1.7)	1.44 (1.30; 1.58)			
*Changes*	−0.44 (−0.52; −0.37)	−0.42 (−0.53; −0.31)	−0.46 (−0.57; −0.36)			
T-C (mg/dl)				0.0003	0.346	0.958
*Baseline*	231.1 (223.8; 238.3)	226.4 (216,8; 236.0)	235.8 (224.8; 246.9)			
*Changes*	−13.3 (−18.5; −8.2)	−11.2 (−17.6; −4.8)	−15.5 (−23.6; −7.3)			
LDL-C (mg/dl)				0.235	0.441	0.688
*Baseline*	146.4 (139.8; 153.0)	143.1 (133.8; 152.4)	149.7 (140.3; 159.3)			
*Changes*	−7.2 (−12.0; −2.4)	−4.9 (−10.2; 0.4)	−9.4 (−17.6; −1.3)			
HDL-C (mg/dl)				0.169	0.182	0.234
*Baseline*	54.2 (52.5; 55.9)	53.1 (50.9; 55.3)	55.3 (52.7; 58.0)			
*Changes*	−1,1 (−2,3; 0.2)	−2.0 (−3.4; −0.7)	−0.1 (−2.3; 2.1)			
TG (mg/dl)				<0.0001	0.607	0.604
*Baseline*	160.8 (145.0; 176.5)	164.1(144.0; 184.3)	157.4 (132.7; 182.1)			
*Changes*	−34.0 (−46.6; −21.5)	−38.8 (−52.3; −25.2)	−33.9 (−55.1; −12.8)			
Hcy (μM)				0.002	0.412	0.776
*Baseline*	11.2 (10.4; 11.9)	10.9 (10.2; 11.6)	11.5 (10.1; 12.8)			
*Changes*	0.7 (0.3; 1.2)	0.8 (0.2; 1.4)	0.7 (0.06; 1.3)			
SBP (mmHg)				<0.0001	0.8024	0.7985
*Baseline*	141.5 (139.0; 144.0)	142.1 (138.9;145.4)	140.9 (137.0; 144.8)			
*Changes*	−9.6 (−11.9; −7.2)	−10.4 (−13.3; −7.4)	−10.2 (−13.7; −6.7)			
DBP (mmHg)				<0.0001	0.946	0.446
*Baseline*	−8.7.0 (85.2; 88.7)	86.9 (84.9; 89.8)	87.0 (84.2; 89.8)			
*Changes*	−6.7 (−8.3; −5.2)	−8.1 (−10.1; −6.1)	−6.7 (−9.0; −4.3)			

Abbreviations: GLU: glucose; INS: insulin; HOMA2-IR: homeostatic model assessment of insulin resistance; T-C: total cholesterol; LDL-C: low-density lipoprotein cholesterol; HDL-C: high-density lipoprotein cholesterol; TG: triglyceride; Hcy: homocysteine; SBP: systolic blood pressure; DBP: diastolic blood pressure; T: time; G: group; T × G: time–group interaction; ^a^Values are expressed as means (95% CI); ^b^Linear mixed model for values at each time point, significant at *P* < 0.05; CED: baseline: n = 72; week 4: n = 70; week 8: 68; week 12: n = 65; week 16: n = 63; MED: baseline: n = 72; week 4: n = 72; week 8: 72; week 12: n = 70; week 16: n = 67; in the table values at baseline and changes from baseline to follow-up are presented.

**Table 3:** Dietary intake characteristics in the CED and MED groups^a^.

Variables	CED group	MED group	*P*-values^b^		
			T	G	T × G
Energy (MJ/day)			<0.0001	0.508	0.968
*Baseline*	8.1 (7.8; 8.4)	8.0 (7.7; 8.3)			
*Changes*	−2.4 (−2.7; −2.1)	−2.4 (−2.8; −2.1)			
Protein (%E)			<0.0001	0.057	0.466
*Baseline*	15.9 (15.3; 16.4)	16.5 (15.9; 17.1)			
*Changes*	2.5 (1.9; 3.1)	2.2 (1.5; 2.9)			
Carbohydrate (%E)			0.013	0.213	<0.0001
*Baseline*	48.6 (47.1; 50.1)	47.5 (45.9; 49.1)			
*Changes*	5.7 (3.9; 4.7)	−2.5 (−4.3; −0.8)			
DF (g/4.2 MJ)			<0.0001	0.631	<0.0001
*Baseline*	11.2 (10.6; 11.9)	11.0 (10.2; 11.8)			
*Changes*	9.1 (8.1; 10.2)	6.1 (5.2; 7.1)			
Fat (%E)			<0.0001	0.847	<0.0001
*Baseline*	35.9 (34.5; 37.3)	36.1 (34.7; 37.4)			
*Changes*	−8.5 (−10.1; −7.1)	0.3 (−1.2; 1.8)			
SFAs (%E)			<0.0001	0.201	0.322
*Baseline*	17.4 (16.6; 18.3)	16.9 (16.1; 17.7)			
*Changes*	−8.6 (−9.5; −7.7)	−8.4 (−9.2; −7.6)			
MUFAs (%E)			0.093	0.094	<0.0001
*Baseline*	13.2 (12.4; 14.1)	14.0 (13.2; 14.8)			
*Changes*	−3.9 (−4.7; −3.0)	5.0 (4.0; 6.0)			
PUFAs (%E)			<0.0001	0.852	0.943
*Baseline*	5.2 (4.8; 5.6)	5.2 (4.7; 5.9)			
*Changes*	3.9 (8.7; 9.5)	3.8 (3.1; 4.4)			

Abbreviations: DF: Dietary fiber; MUFAs: monounsaturated fatty acids; SFAs: saturated fatty acids; PUFAs: polyunsaturated fatty acids; T: time; G: group; T × G: time–group interaction; ^a^Values are expressed as means (95% CI); ^b^Linear mixed model for values at each time point, significant at *P* < 0.05; CED: baseline: n = 72; week 4: n = 70; week 8: 68; week 12: n = 65; week 16: n = 63; MED: baseline: n = 72; week 4: n = 72; week 8: 72; week 12: n = 70; week 16: n = 67; in the table values at baseline and changes from baseline to the average of the four postrandomization time points are presented.

### Changes in physical activity and metabolic measurements

The reported RMR, npRQ, and PA data are given in Table 4. At baseline, there were no significant differences between the groups in terms of these variables. At all time points, the mean RMR values were significantly lower (*P* < 0.001) than at baseline, with no difference between the groups (*P* = 0.881, interaction effect; Table 4). Since FFM is known to be the predominant determinant of RMR, the association between changes in both parameters was considered. In the CED group, though not in MED, the reduction in RMR correlated significantly with the decreases in FFM (r = 0.37, *P* < 0.001). Compared to the MED, the CED group reported higher npRQ values (*P* = 0.017, interaction effect). No changes in the pattern of PA were seen over the course of the dietary intervention (Table 4).

**Table 4:** PA and metabolic measurements in the CED and MED groups.

Variables	CED group	MED group	*P*-values^c^		
			T	G	T × G
RMR (MJ/day)^a^			<0.001	0.438	0.881
*Baseline*	6.2 (6.1, 6.4)	6.3 (6.1, 6.5)			
*Changes* ^e^	−0.3 (−0.4, −0.1)	−0.2 (−0.4, −0.1)			
NpRQ^a^			<0.001	0.631	0.017
*Baseline*	0.85 (0.84, 0.86)	0.85 (0.84, 0.86)			
*Changes* ^e^	0.005 (0.02, 0.01)	−0.03 (−0.04, −0.01)			
<600MET/min/week (n)^b^			0.779^d^		
*Baseline*	16.0	14.0			
*Average* ^f^	19.0	18.0			
600 to 1499MET/min/week (n)^b^			0.728^d^		
*Baseline*	35.0	31.0			
*Average* ^f^	36.0	28.0			
≥1500MET/min/week (n)^b^			0.847^d^		
*Baseline*	21.0	27.0			
*Average* ^f^	17.0	26.0			

Abbreviations: ^a^Continuous values are expressed as means (95% CI) and ^b^categorical values as number of subjects; RMR: resting metabolic rate; npRQ: nonprotein respiratory quotient; ^c^Linear mixed model for values at each time point, significant at *P* < 0.05; ^d^difference between number of subjects in the compared groups (Chi-square test of independence); CED: baseline: n = 72; week 4: n = 70; week 8: 68; week 12: n = 65; week 16: n = 63; MED: baseline: n = 72; week 4: n = 72; week 8: 72; week 12: n = 70; week 16: n = 67; values at baseline and ^e^changes from baseline to follow-up, as well as the ^f^average number of subjects in each PA category averaged across the four postrandomization time points are presented.

### Changes in energy and selected dietary macronutrient intakes according to dietary adherence

Perceived hunger levels were significantly elevated over the trial (*P* < 0.001), ranging from 32.5 to 45.5 and from 32.9 to 45.9 in the CED and the MED group respectively, with no difference between the groups (*P* = 0.990, interaction effect). Adherence to the prescribed diets was significantly reduced over the course of trial (*P* < 0.001), ranging from 1.76 to 2.38 and from 1.92 to 2.48 in the CED and the MED groups, respectively with no difference between the groups (*P* = 0.189, interaction effect). Total energy and selected dietary macronutrient intakes were calculated for the more and less adherent participants (Table 5). In both diet groups, the more adherent participants had a significantly lower (*P* < 0.001) mean energy intake (CED: 5.52 MJ; 95% CI: 5.40, 5.65; MED: 5.91 MJ 95% CI: 5.78, 6.04) than those in the low adherence groups (CED: 5.37 MJ 95% CI: 5.22, 5.52; MED: 5.8 MJ 95% CI: 5.65, 5.96). Almost all reported macronutrient intakes (apart from the percentage energy derived from PUFAs) significantly differed between the more and the less adherent participants from the CED group. Participants who were more adherent to the MED reported a significantly lower (*P* = 0.40) energy intake from SFAs than their less adherent counterparts. Also in the MED group, the energy intake from fat (*P* = 0.020) and MUFAs (*P* = 0.001) was significantly higher in more adherent than in less adherent participants.

**Table 5:** Average energy and macronutrient intakes across median adherence in both diets^a^.

Variables	CED group		*P*-value^b^	MED group		*P*-value^b^
	High adherence	Low adherence		High adherence	Low adherence	
Energy (MJ)	5.52 (5.40; 5.65)	5.91 (5.78; 6.04)	<0.001	5.37 (5.22; 5.52)	5.80 (5.56; 5.96)	<0.001
Protein (%E)	17.8 (17.3; 18.2)	19.0 (18.5; 19.5)	<0.001	18.3 (17.8; 18.9)	19.1 (18.6; 19.6)	0.105
Carbohydrate (%E)	56.1 (55.2; 57.1)	52.4 (51.5; 53.4)	<0.001	44.3 (43.0; 45.5)	45.6 (44.3; 47.0)	0.080
Total fat (%E)	26.1 (25.3; 26.9)	28.6 (27.8; 29.4)	<0.001	37.4 (36.2; 38.7)	35.3 (34.0; 36.6)	0.020
SFAs (%E)	7.9 (7.4; 8.5)	9.8 (9.2; 10.4)	0.002	8.1 (7.6; 8.6)	8.8 (8.3; 9.4)	0.040
MUFAs (%E)	8.8 (8.4; 9.3)	9.9 (9.4; 10.4)	<0.01	20.0 (19.1; 20.8)	17.9 (17.1; 18.8)	0.001
PUFAs (%E)	9.3 (8.7; 9.9)	8.8 (8.3; 9.4)	0.264	9.3 (8.7; 9.9)	8.5 (7.9; 9.1)	0.080
DF (g/4.2MJ)	23.2 (22.5; 23.7)	18.1 (16.1; 19.2)	<0.001	17.2 (16.4; 18.0)	17.0 (16.2; 17.8)	0.762

Abbreviations: ^a^Values are expressed as means (95% CI); SFAs: saturated fatty acid; MUFAs: monounsaturated fatty acids; PUFAs: polyunsaturated fatty acids; DF: dietary fiber; ^b^comparison of between-group (high vs. low adherence) changes (from baseline to average across the four postrandomization time points); High adherence here means adherence scores above a median value, with low adherence referring to adherence scores below that median value.

### Changes in anthropometric, lipid, and nonlipid parameters according to dietary adherence

**Figure 2:**
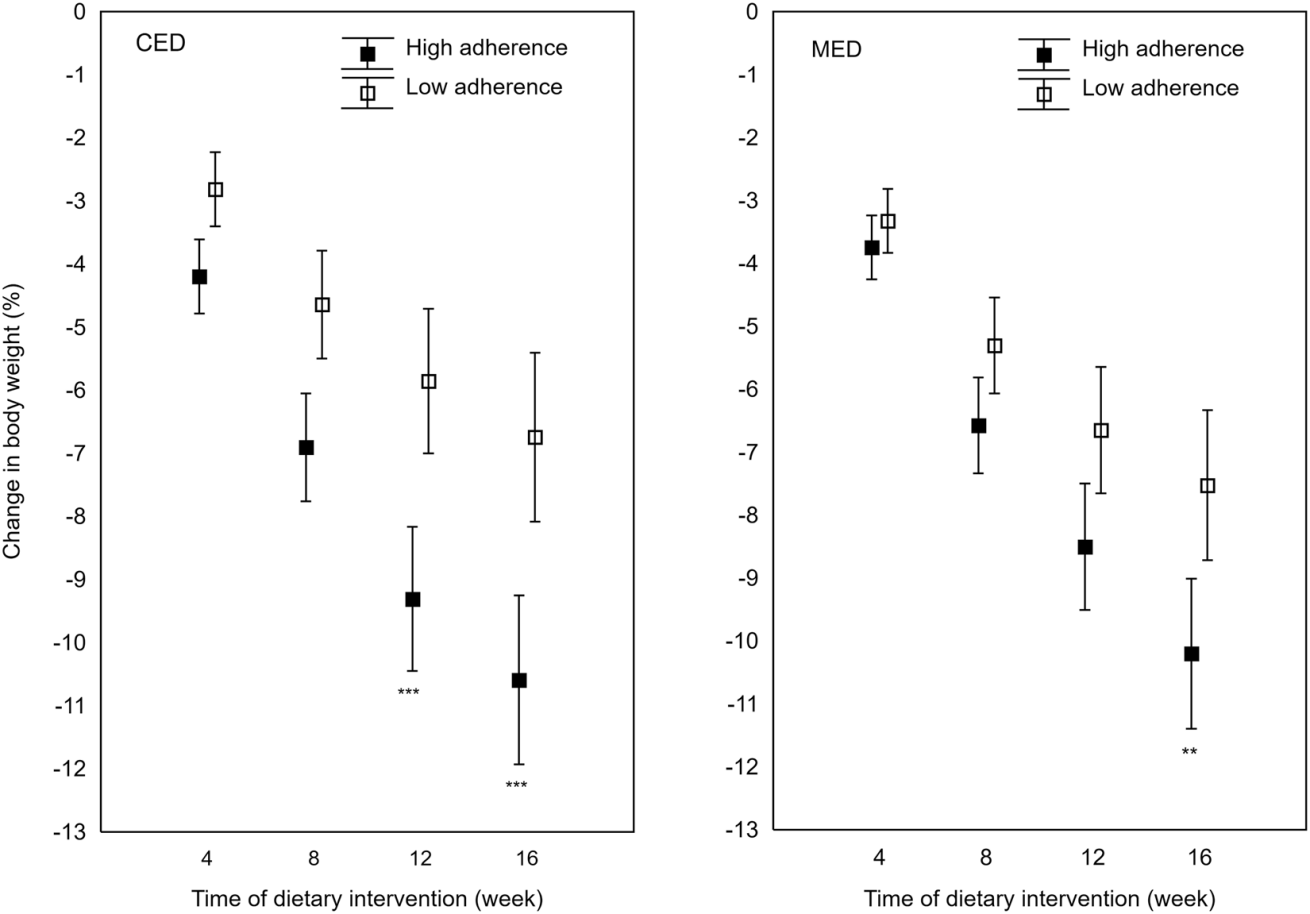
Mean percent body weight changes over 16 weeks of dietary intervention in the more adherent (filled square) and less adherent (open square) postmenopausal women from the CED (left side) and the MED (right side) groups, respectively. Data are presented as means (CI 95%), **P < 0.01, ***P < 0.001 for differences between level of adherence at respective time points.

It was seen that adherence to the diets affected the clinical parameters (Table 6). Within each diet group, the more adherent participants lost more weight (CED: −9.0 kg; 95% CI: −10.2, −7.8; MED: −9.1 kg; 95% CI: −10.2; −8.0; *P* < 0.001), than the less adherent counterparts (CED: −5.9 kg; 95% CI: −7.1, −4.7; MED: −6.3 kg 95% CI: −7.3, −5.2). Significant differences in body weight loss, expressed as a percentage of initial body weight between high and low adherent participants, were found at 12 and 16 week of the trial in the CED and the MED groups, respectively (Fig. 2). Also, the reductions in WC, FM, and FFM were higher among the more adherent participants, regardless of the diet assigned. However, only the more adherent women from the CED group lost significantly more VF (−0.30 kg; 95% CI: −0.34, −0.26, *P* = 0.010) than the less adherent counterparts (−0.23 kg; 95% CI: −0.27, −0.19). Among the more adherent participants from both study groups, a tendency for improvement was only seen in certain metabolic syndrome factors: in the CED group, mainly in GLU, INS, HOMA2-IR, T-C, LDL-C, and TG, while in the MED group in T-C, HDL-C, and TG.

**Table 6:** Comparison of changes in selected parameters across median adherence in both diets^a^.

Variables	CED group		*P*-value^b^	MED group		*P*-value^b^
	High adherence	Low adherence		High adherence	Low adherence	
Δ Body weight (kg)	−9.0 (−10.2; −7.8)	−5.9 (−7.1; −4.7)	<0.001	−9.1 (−10.2; −8.0)	−6.3 (−7.3; −5.2)	<0.001
Δ WC (cm)	−9.2 (−10.5; −7.8)	−5.8 (−7.2; −4.5)	<0.001	−8.4 (−9.5; −7.2)	−6.4 (−7.5; −5.3)	0.014
Δ FM (kg)	−7.5 (−8.4; −6.5)	−5.8 (−6.8; −4.9)	0.020	−7.3 (−8.2; −6.4)	−5.8 (−6.7; −4.9)	0.020
Δ FFM (kg)	−1.5 (−2.1; −1.0)	−0.1 (−0.6; 0.5)	<0.001	−1.8 (−2.4−1.2)	−0.5 (−1.0; 0.1)	0.002
Δ VF (kg)	−0.30 (−0.34; −0.26)	−0.23 (−0.27; −0.19)	0.010	−0.27 (−0.32; −0.22)	−0.23 (−0.28; −0.18)	0.285
Δ GLU (mg/dl)	−6.5 (−9.5; −3.6)	−4.2 (−7.2; −1.3)	0.272	−5.6 (−8.4; −2.7)	−7.2 (−10.0; −4.3)	0.432
Δ INS (µU/ml)	−3.7 (−4.8; −2.5)	−2.7 (−3.9; −1.5)	0.256	−4.0 (−5.1; −2.9)	−3.3 (−4.4; −2.2)	0.397
Δ HOMA2-IR	−0.50 (−0.67; −0.34)	−0.38 (−0.53; −0.23)	0.257	−0.38 (−0.53; −0.23)	−0.43 (−0.65; −0.27)	0.456
Δ T-C (mg/dl)	−14.4 (−23.5; −5.3)	−8.0 (−17.1; 1.1)	0.319	−17.2 (−28.8; −5.6)	−13.8 (−25.3; −2.2)	0.676
Δ LDL-C (mg/dl)	−6.5 (−14.0; 1.04)	−3.3 (−10.8; 4.2)	0.551	−9.0 (−20.6; 2.5)	−9.8 (−21.4; 1.7)	0.922
Δ HDL-C (mg/dl)	−2.2 (−4.1; −0.3)	−1.9 (−3.7; 0.01)	0.787	0.9 (−2.2; 4.0)	−1.0 (−4.1; 2.1)	0.400
Δ TG (mg/dl)	−42.6 (−61.8; −23.4)	−35.0 (−54.3; −15.7)	0.581	−50.9 (−80.5; −21.3)	−16.9 (−46.5; −12.7)	0.110
Δ Hcy (μM)	0.9 (0.01; 1.7)	0.8 (−0.05; 1.7)	0.924	0.9 (0.1; 1.8)	0.4 (−0.5; 1.2)	0.367
Δ SBP (mmHg)	−10.8 (−15.0; −6.6)	−10.0 (−14.2; −5.8)	0.791	−10.3 (−15.3; −5.4)	−10.0 (−15.0; −5.1)	0.938
Δ DBP (mmHg)	−8.7 (−11.5; −5.8)	−7.4 (−10.3; −4.6)	0.546	−7.4 (−10.8; −4.1)	−5.9 (−9.2; −2.6)	0.519

Abbreviations: ^a^Values are expressed as means (95% CI); BW: body weight; WC: waist circumferences; FM: fat mass; FFM: fat-free mass; VF: visceral fat; GLU: glucose; INS: insulin; HOMA2-IR: Homeostatic Model Assessment of Insulin Resistance; T-C: Total cholesterol; LDL-C: low-density lipoprotein cholesterol; HDL-C: high-density lipoprotein cholesterol; triglyceride: TG; Hcy: homocysteine; SBP: systolic blood pressure; DBP: diastolic blood pressure; ^b^values for comparison of between-group (high vs. low adherence) changes (from baseline to follow-up). High adherence here means adherence scores above a median value, with low adherence referring to adherence scores below that median value.

**Table 7:** Correlation between visceral fat and energy and macronutrient intake at baseline, and changes in visceral fat and caloric deficit, as well as changes in macronutrient intake.

Dietary variables	Baseline		Changes in dietary variables	CED group		MED group	
	r	*P*		r	*P*	r	*P*
	Visceral fat			Changes in visceral fat from baseline			
Energy intake (MJ)	−0.02	0.851	Δ Energy intake (MJ)	0.17	0.166	0.24	0.044
Carbohydrate (%E)	−0.18	0.030	Δ Carbohydrate (%E)	−0.25	0.033	0.14	0.239
Fat (%E)	0.21	0.010	Δ Fat (%E)	0.24	0.043	−0.10	0.408
SFA (%E)	0.17	0.025	Δ SFA (%E)	0.27	0.020	−0.13	0.261
MUFA (%E)	0.13	0.118	Δ MUFA (%E)	0.19	0.110	−0.01	0.905
PUFA (%E)	0.13	0.111	Δ PUFA (%E)	−0.09	0.436	−0.06	0.637
DF (g/4.2MJ)	−0.08	0.346	Δ DF (g/4.2MJ)	−0.25	0.031	0.05	0.702

Abbreviations: SFAs: saturated fatty acids; MUFAs: monounsaturated fatty acids; PUFAs: polyunsaturated fatty acids; DF: Dietary fiber; Pearson correlation coefficient analysis was performed.

### Effects of diet type on visceral fat reduction

At baseline, VF was correlated with the percentage energy from total (r = 0.21, *P* = 0.010) and saturated fat (r = 0.17, *P* = 0.025), and negatively with percentage energy from carbohydrates (r = −0.18, *P* = 0.030, Table 7). In the CED group, the reduction in this parameter correlated with an increase in the proportion of carbohydrates (r = −0.25, *P* = 0.033) and DF (r = −0.25, *P* = 0.031), and decreases in the total (r = 0.24, *P* = 0.043) and saturated (r = 0.27, *P* = 0.020) fat. In the MED group, changes in VF content correlated with energy intake (r = 0.17, *P* = 0.044, Table 7).

## Discussion

In our study, postmenopausal women with central obesity and with at least one another MetS criterion were randomly assigned to one of two types of energy-restricted diets—to the CED or to the MED, which had different contents of carbohydrates, DF, total fat, and MUFAs. We found that the postmenopausal women lost on average 7.6 kg weight (95% CI; −8.2, −7.0), 7.4 cm WC (−8.1, −6.8), 6.6 kg FM (−7.1, −6.1), and 0.26 kg VF (−0.28, −0.23), with no differences between diets. The reductions in BW were accompanied by beneficial changes in the concentrations of some blood metabolic biomarkers, such as GLU, INS, HOMA2-IR, T-C, TG, and blood pressure. Neither the CED nor the MED significantly improved LDL-C, HDL-C, or Hcy concentrations. In general, it could be concluded that both reduced-calorie diets were equally efficient regardless of their macronutrient compositions. The findings of our study are consistent with existing data showing that energy restriction diets with different macronutrient compositions can lead to weight loss and reduction of MetS risk^25–28^. For example, an energy-restricted MED^29^ or the low fat, hypocaloric diet used in the Look Ahead Trial^26^ both have a beneficial effects on weight loss and improvement of MetS risk factors. However, none of these studies determined whether the metabolic effect could be attributed to the weight loss or to the diets themselves.

The weight loss observed in our study, although satisfying, was less than we had expected, taking into account the targeted energy deficit (2.93 MJ). It seems that individual adherence to dietary goals could be the primary determinant of the diet efficacy. Regarding this, Gibson & Sainsbury indicated that the level of adherence may be influenced by how different a dietary intervention is from a person’s usual diet^30^. We therefore assumed that the CED may be a more feasible diet option than the MED in Central Europe. However, we observed that the adherence score declined similarly over time in both groups. Feelings of hunger are often cited as the reason for poor adherence to energy-restricted weight loss regimes^31^. In our study, subjective sensations of hunger were similarly elevated in both groups over the course of the trial. It was found that substantially diminished adherence after the first few months is typical in weight-loss trials, and this suggests that participants in weight-loss programs revert to their customary macronutrient intakes over time^25^. Indeed, less adherent postmenopausal women from the CED and MED groups tended to eat respectively more fat and more carbohydrate. Comparisons between more and less adherent postmenopausal women were also made in relation to changes in several anthropometric and metabolic parameters. Within each diet group, the more adherent participants from the CED and MED groups lost significantly more weight by respectively 3.1 kg (34.4%) and 2.8 kg (29.7%) than the less adherent counterparts. A greater reduction in body weight observed in the more adherent participants from both study groups was associated with a greater reduction in WC and FFM.

However, in the CED group only, the more adherent participants lost significantly more VF (by 23.3%) than their less adherent counterparts. In the MED group, the nonsignificant difference in VF reductions between the more and the less adherent participants was only 14.8%. Because VF is closely related to the metabolic consequences of obesity^32^, the effect of dieting on the reduction of this type of fat is the most desirable. Only few trials have directly measured changes in VF in response to weight-reducing diets. There is thus still a debate on which, if any, diet most effective for VF loss^33^. We found that in the CED group, the reduction in VF was associated with increases in the proportion of carbohydrate and DF intake, and with decreases in the total and SFAs intake. Our findings are consistent with the results of previous studies showing that overeating SFAs promotes VF storage^34^. In the case of DF, it was reported that increases in DF intake over a two-year period were associated with less VF accumulation in overweight Latino youth^35^. The mechanism for how this dietary compound might affect visceral adiposity is unknown; however, it was found that DF increased fecal bulk, decreased transit time, and increased insulin sensitivity^35^. In our study, the postmenopausal women who were more adherent to the CED had higher (by ~5 g/4.2 MJ) intake of DF than their less adherent counterparts. Anderson & Gustafson indicated that high dietary fiber intake may independently enhance adherence to the prescribed diet, perhaps by increasing satiety or because of the simplicity of the concept^36^. Indeed, we also observed that higher intake of dietary fiber was associated with higher adherence level in the CED group (r = −0.44, *P* < 0.001; data not shown in the results section), but not in the MED (r = 0.10, *P* = 0.887; data not shown in the results section). The DF in the CED was mostly in soluble form and was derived mainly from oats, barley, apples, plums, beetroot, and flaxseeds. The proportion of soluble to insoluble DF in this type of diet was 35% to 65%, while in the MED this was only 20 to 80%. Schneeman reported that 30–50% soluble and 50–70% insoluble DF are considered to be well-balanced proportions for optimum health benefits^37^. Moreover, a portion of oats or barley with seasonal fruits and ground flaxseeds consumed by the postmenopausal women in the CED group at breakfast provided ~5 g β-glucan—an important food component in the modulation of metabolic dysregulations associated with metabolic syndrome^38^.

Surprisingly, in the MED study group, the reduction in VF was only due to the energy deficit. In their review, Bendall *et al*. considered to whether the reductions in central obesity reported in several studies resulted from the MED *per se*, or simply from the energy restriction accompanying this dietary regimen^39^. They concluded that the observed greater reduction in central obesity could be explained by the higher postprandial fat oxidation rate and the higher thermic effect following meals high in MUFAs^40^. In our study, MED significantly affected postabsorptive npRQ levels, tending them towards lower values. These results seem to reflect the dietary composition of the MED. Furthermore, it appears that oleic acid (the predominant MUFAs in olive oil) is more favorably oxidized, while saturated fats are more favorably stored^30^. Indeed, in our study also, significant correlations were seen between VF deposits and the percentage of total fat and SFAs at the beginning of the trial.

This study has several strengths. First, the study was conducted in an established, short-term, and successful randomized trial. Second, the groups were homogenous, postmenopausal women who, apart from central obesity, had at least one feature of MetS. There was a strong support from dieticians throughout the intervention period, and the participants received not only detailed dietary plans, but also ready-to-eat main meals. There are also some limitations to the finding reported here. First, even if the sample size (72 postmenopausal women in each dietary arm) was large enough to detect differences in body weight between the two tested diets over 16 weeks of dietary intervention, 36 participants in each more and less adherent subgroup may be inadequate to detect significant differences in some metabolic syndrome risk factors. Second, the duration of our trial was only 16 weeks, which might have been too short to detect the minor effects of the diets. However, given that there were significantly lower body weight losses at week 12 of the trial in the less adherent women in the CED group, it seems that a longer trial might have been ineffective. Third, deviation from macronutrient goals as a measure of adherence was heavily dependent on self‐reported dietary recall. In future studies, other more sensitive biochemical indicators (such as erythrocyte membrane fatty acid composition) should be used to monitor adherence. Fourthly, the adherence levels observed in this study are likely even higher than in the general population, which may in part be explained by the fact that main meals were provided every day to the participants for free. In future, the effectiveness of both diets should be tested in real world settings.

## Conclusion

The short-term dietary treatment using the CED or the MED was associated with similar improvements in some anthropometric, lipid, and nonlipid parameters; however, adequate adherence to the prescribed diet is important in weight loss success and in achieving improvements in metabolic health. Apart from adherence, substantial differences in the proportions of dietary macronutrients also play an important role in visceral fat loss success, and this success is possible when adequate DF is provided.

## Electronic supplementary material

Consort 2010 checklist

Project summary WHO
