# Author Correction: Weight loss and metabolic health effects from energy-restricted Mediterranean and Central-European diets in postmenopausal women: A randomized controlled trial

**DOI:** 10.1038/s41598-019-51387-3

**Published:** 2019-10-31

**Authors:** Joanna Bajerska, Agata Chmurzynska, Agata Muzsik, Patrycja Krzyżanowska, Edyta Mądry, Anna M. Malinowska, Jarosław Walkowiak

**Affiliations:** 1 Institute of Human Nutrition and Dietetics, Poznan University of Life Sciences, Wojska Polskiego 31, 60–624 Poznan, Poland; 2 First Subdepartment of Pediatrics, Department of Pediatric Gastroenterology and Metabolism, Poznan University of Medical Sciences, Szpitalna 27/33, 60-572 Poznan, Poland

Correction to: *Scientific Reports* 10.1038/s41598-018-29495-3, published online 24 July 2018

The Acknowledgements section in this Article is incomplete.

“We thank the subjects for their committed participation in this study. The project was financed by a National Science Centre award based on decision number DEC-013/09/B/NZ9/02365.”

should read:

“We thank the subjects for their committed participation in this study. The project was financed by a National Science Centre award based on decision number DEC-2013/09/B/NZ9/02365.”

