# Supplementary material for: Weight loss and metabolic health effects from energy-restricted Mediterranean and Central-European diets in postmenopausal women: A randomized controlled trial
Source: Sci Rep. 2018 Jul 24;8:11170. doi: 10.1038/s41598-018-29495-3 (PMC6057942; doi:10.1038/s41598-018-29495-3)
Supplement: Supplementary file 2 — Project summary WHO [file 41598_2018_29495_MOESM2_ESM.docx]

**Part 1**

**I. Project summary.** Improvement of metabolic health is the cornerstone of obesity therapy in postmenopausal women. One dietary model for improving metabolic health is a low-calorie, low-fat diet. Another dietary model is the Mediterranean diet (MED). However, it was stated that adoption of the MED by other than Southern Europe populations is difficult. At the same time, interest has increased in diets based on local healthy food items in the prevention or treatment of nutritionally dependent diseases. Therefore, the following hypotheses will be verified: 1) implementation of traditional and health-promoting food items available in Central Europe into the diet of centrally obese postmenopausal women (COPW) can be as effective in weight loss and improvement of selected risk factors for metabolic syndrome (MetS) as the MED is; 2) among COPW the effectiveness of a both dietary intervention depends on various factors, such as polymorphism in genes responsible for body weight (BW) control and lipid metabolism; 3) components of both diets can influence the gene-expression of selected inflammatory cytokines. Nonsmoking, COPW will be randomly assigned to the MED or the Central European diet (CED) for 16 weeks. Changes in BW, body composition, waist circumference will be measured in four-week intervals. Additionally, dietary intake, blood pressure and physical activity level will be checked. Before and after dietary intervention changes in total cholesterol, LDL-cholesterol, HDL-cholesterol, triglycerides, glucose, insulin, homocysteine as well as inflammatory markers will be done. In the same time influence of the CED and the MED on visceral fat content, liver function, resting metabolic rate, gene expression of inflammatory cytokines will be done. To check associations between genes polymorphism and effectiveness of the dietary intervention and metabolic variables, the COPW will be genotyped for FTO, PPAR-γ and IL-6. A year after the completion of the experiment BW change and changes in risk factors of MetS will be done.

**II. General information**

**1. Protocol title, protocol identifying number (if any), and date.**

The impact of Central European diet on metabolic risk factors - a modern nutrition approach. Duration of the project 2014-2018

**2. Name and address of the sponsor/funder.**

National Science Centre, ul. Królewska 57, 30-081 Kraków, Poland

Telephone: +48 12 341 90 00, Fax: +48 12 341 90 99, E-mail: biuro@ncn.gov.pl

**III. Name and title of the investigator(s) who is (are) responsible for conducting the research, and the address and telephone number(s) of the research site(s), including responsibilities of each.**

**1. Dr. Joanna Bajerska, PhD; PI**

Institute of Human Nutrition and Dietetics, Poznań University of Life Sciences, Wojska Polskiego 31, 60-624 Poznań, Poland

Telephone: +48618466056, Fax: +48618487332, E-mail: [joanna.bajerska@up.poznan.pl](mailto:joanna.bajerska@up.poznan.pl)

**2. Agata Chmurzyńska, PhD; Co-PI**

Institute of Human Nutrition and Dietetics, Poznań University of Life Sciences, Wojska Polskiego 31, 60-624 Poznań, Poland, Telephone: +486184661816, Fax: +48618487332, E-mail: joanna.bajerska@up.poznan.pl

**3. Prof. Jarosław Walkowiak, PhD, MD; Co-PI**

Department of Pediatric Gastroenterology and Metabolism, Poznań University of Medical Sciences, Szpitalna 27/33, 60-572 Poznań, Telephone: +48618491432; Fax: +48618472685, E-mail: jarwalk@ump.edu.pl

**The PI/Co-PI are responsible for:**

• preparing, conducting, and and administration of a research grant

• ensuring that the subjects’ rights, safety, and welfare are protected

• overseeing the informed consent process

• supervising and educating staff involved in the clinical trial

• providing records for inspection

• complying with record-keeping

• overseeing the integrity and analysis of research data

• ensuring that protocols are followed and that data are collected promptly and accurately

• ensuring necessary approvals are obtained

**IV. Name(s) and address(es) of the clinical laboratory(ies) and other medical and/or technical department(s) and/or institutions involved in the research**

1. Molecular Metabolism Laboratory, Institute of Human Nutrition and Dietetics, Poznań University of Life Sciences, Wojska Polskiego 31, 60-624 Poznań, Poland

2. Department of Pediatric Gastroenterology and Metabolism, Poznań University of Medical Sciences, Szpitalna 27/33, 60-572 Poznań

**Rationale & background information.** Menopause is a critical period in a woman’s life, during which body weight gain and redistribution of adipose tissue towards a more central/android phenotype has been observed ^1^. There is increasing evidence that visceral obesity is linked to metabolic risk factors of cardiovascular diseases and diabetes ^1, 2^. Successful strategies for reducing overweight and improving metabolism in postmenopausal women are therefore of the utmost importance ^3^. One dietary model for reducing the incidence of cardiovascular events, which is recommended by the US National Cholesterol Education Program (NCEP)^4^ and the American Heart Association (AHA)^5^, is a low-calorie, low-fat diet. Another dietary model, the so-called Mediterranean diet (MED) is characterized by a relatively high fat intake (up to 40% of total daily calories), of which monounsaturated fatty acids (MUFAs) give 15%–25% of the energy ^6,7^. The benefits of the MED for body weight loss and for reducing the incidence of cardiovascular events have been pointed out in many studies, where both energy-restricted and *ad libitum* approaches have been applied ^8,9,10,11,12^. However, Papadaki & Scott have stated that adoption of the MED by other than Southern Europe populations is difficult, on account of its high cost and the limited availability of these unfamiliar food items, as well as cultural differences that affect food choice ^13^. At the same time, interest has increased in diets based on local healthy food items in the prevention or treatment of nutritionally dependent diseases. Eating a traditional balanced Nordic diet has been associated with lower death rates in a Danish cohort study ^14^. Similarly to the Nordic region, in the central part of Europe (such as Poland), there are several common food items—including fish (herrings), whole grain rye (eaten as rye bread), oats (eaten as oatmeal), root vegetables (beetroot), cabbages, berries, apples, and plums—which have much better nutrient profiles than the typical Western diet. The aim of this study was thus to compare the effectiveness of two energy-restricted diets differing in macronutrients on weight loss and improvement in selected risk factors for metabolic syndrome (MetS). The diets used were the Mediterranean diet (MED), which is moderate in fat and has a high proportion of MUFAs, and the so-called Central European diet (CED), which is low in fat, moderate in carbohydrates, and high in dietary fiber derived from central European food items. Since the outcomes of dietary interventions may depend greatly on adherence to the diet, we considered how this factor contributed to the effects of the CED or the MED.

#### Study goals and objectives. The project will include the following objectives:

- description of dietary intervention including food items characteristic for MED and CED;
- implementation of a 16-wk dietary intervention program including the MED or CED diet (randomized controlled trial) in centrally obese postmenopausal women with at least one other criterion of MetS;
- the assessment of effectiveness of both dietary interventions in terms of changes in body weight, visceral fat, as well as changes in MetS risk factors (WC, systolic blood pressure; SBP, diastolic blood pressure; DBP, fasting glucose; GLU, high-density lipoprotein cholesterol; HDL-C, triglyceride; TG), biomarkers for inflammation at 16 weeks from baseline measurements. The influence of dietary components on the gene-expression of selected inflammatory cytokines will be measured.
- checking frequencies of selected polymorphic alleles associated with lipid metabolism or influencing the effectiveness of the proposed dietary intervention in subjects enrolled in the study;
- the assessment of long-term effects on nutritional habits, weight maintenance and visceral obesity-associated abnormalities, such as blood lipid or glucose levels (one year after the end of the dietary intervention).

**Study Design**

The study will be involved a 16-week, two-arm parallel group, randomized controlled trial conducted in Poznan University of Life Sciences and Poznań University of Medical Sciences. Nonsmoking, postmenopausal women (with absence of menses > 12 months or a serum follicle-stimulating hormone >30 IU/mL) with central obesity (waist circumference; WC ≥ 80 cm), plus at least one other criterion of metabolic syndrome (MetS) ^15^, who wished to lose weight, will be recruited through advertisements. Women with type 2 diabetes, monogenic dyslipidemia, history of cardiovascular disease, using hypoglycemic, hypolipidemic, anti-inflammatory and weight loss agents as well as any drug known to influence liver function, with endocrine disorders or on hormonal replacement therapy will be not eligible. Exclusion criteria also will be included significantly weight changes in the six months prior to the current study, intolerance or food allergy to key components of the intervention diets, excessive alcohol consumption (>2 drinks/day). The potential participants will be screened during an interview to determine eligibility to the study. Postmenopausal women fulfilled inclusion criteria will be randomly assigned (1:1) to the MED or the CED. A computer program will be used to generate the block randomisation sequence (block size 4) with body mass index as the stratification factor.

**Methodology**

**Dietary intervention.** Individuals will be fed their assigned supervised diet with a calorie level deficit of ∼700 kcal/day compared to individual energy requirements (estimated by multiplying RMR by an appropriate PA factor). The MED group will be followed a food plan designed on the basis of the Mediterranean dietary recommendations released in 2010 by the Mediterranean Diet Foundation ^6^. To build this menu, typical Mediterranean food products will be used. This MED will be provided approximately 37% energy from total fat, 20% from MUFAs, 9% from polyunsaturated fatty acids (PUFAs), 8% from saturated fatty acids (SFAs), 18% from protein, and 45% energy from carbohydrates. Olive oil will be used in every meal and five to seven nuts will be served once a day. The CED will be based on the recommendations the NCEP ^4^ and the AHA ^5^, and will be designed to provide 27% energy from total fat, 10% from MUFAs, 9% from PUFAs, 8% from SFAs, 18% from protein, and 55% energy from carbohydrate, with a special emphasis on high levels of dietary fiber derived from food items typical of the central European region: cereals (oatmeal, barley, buckwheat, and millet), pulses (peas and beans), vegetables (root vegetables, cruciferous vegetables), and fruits (apples, plums). Added salt and refined fats, as well as sugar, will be excluded from both diets. Fourteen-day cyclic dietary plans will be formulated for both diets using Dietetyk dietary analysis software (Jumar, Poznań, Poland). During the entire intervention period, study participants will be picked up packaged main meals (covering ~35% daily energy requirements) prepared according to a trained dietician’s recipes by a catering company (Passion Łukasz Bakowski, Poznań, Poland). Others meals will be prepared by the study participants themselves, according to the prescribed dietary plan, including recipes and written instructions to facilitate preparation of meals at home. The participants will be recorded any foods that will be consumed in addition to the study meals, and any foods from the study meals that will be not consumed, on a daily compliance questionnaire that will be monitored by study staff. Throughout the intervention, volunteers will be advised to maintain their usual level of PA and keep other lifestyle factors unchanged.

**Measurements.**

**Measure of adherence.** Dietary adherence will be calculated using a Mahalanobis distance equation, which can be used to measure the degree of deviation from the prescribed and actual dietary intake. Details of adherence measuring have been published elsewhere^16^.

**Anthropometric and blood pressure measurements.** Height will be measured to the nearest 0.1 cm (WPT 200.OC). Body weight will be measured to the nearest 0.1 kg with subjects in bathing suits after an overnight fast using a calibrated scale included in the Bod Pod apparatus. Waist circumference (WC) will be measured at the midpoint between the lowest rib and the top of the iliac crest using nonelastic tape. This measurement will be performed twice by a single evaluator. Body composition will be assessed by Bod Pod and DXA. Blood pressure will be measured using a sphygmomanometer.

**Biochemical parameters.** A 12-hour fasting blood samples will be collected before (week 0) after dietary intervention (week 16) as well as one year after dietary intervention. Total cholesterol (T-C), high-density lipoprotein cholesterol (HDL-C), glucose (GLU), and triglyceride (TG) concentrations will be determined in human serum using a Beckman Coulter AU analyzer. Low-density lipoprotein cholesterol (LDL-C) concentrations will be calculated using Friedewald formula^18^. Serum insulin will be determined utilizing chemiluminescence immunoassay, method. HOMA-IR will be calculated according to the formula described elsewhere ^19^. Homocysteine (Hcy) concentrations will be measured in serum samples using HPLC^20^. TNF-α, hsCRP, leptin and IL-6 will be assayed by specific commercially available, enzyme-linked (ELISA) assay kits in accordance with the manufacturer's protocols,

**Dietary assessment.** Dietary intake at baseline and during each control visit will be assessed using a three-day food diary in which the participants were clearly instructed to record information on nonconsecutive days (two weekdays and one weekend day) regarding their food and beverage intake, using household measures. Participants will be provided details of food product brand names, food preparation methods, and any recipes used. The food and beverage quantities obtained from the three-day food diary will be converted into grams and millilitres and computed using the Dietetyk software.

**Resting metabolic rate and respiratory quotient analysis.** RMR and npRQ will be measured after a twelve-hour fast by indirect calorimetry for 30 minutes (10 minutes of acclimatization and 20 minutes of measurements) while the participants will be fasting. Concentrations of CO_2_ and O_2_ will be measured using the ventilated hood technique using a Quark RMR (Cosmed, Rome, Italy).
**Physical activity (PA).** PA will be assessed with the short form of the International Physical Activity Questionnaire (IPAQ-SF). This measure was selected to assess the degree to which participants conformed to the requirement that they not alter their exercise habits during the study, rather than to precisely estimate energy expenditure. The PA results were expressed as metabolic equivalent (MET) minutes per week and classed as inactive (< 600 MET/min/week), moderately active (from 600 to 1499 MET/ min/week), or active (≥ 1500 MET/min/week).

**Determination of attitude toward eating.** To determine whether the subjects’ attitude toward food intake will be changed at any point during the experiment, the three factor eating questionnaire (TFEQ) will be used.

**Self-reported hunger.** The level of hunger was compiled at each of the four post-randomization points using visual analog scales (VAS). Participants reported their level of hunger by marking a point on a 100 mm scale with end descriptors ranging from “Not at all” to “Extremely”.

**Assessment of liver function.** Liver metabolism will be determined by the 13C-methacetin-breath test. Each subject will be received 75 mg methacetin dissolve in 200 ml water. The (13)C-methacetin-breath test will be performed after overnight fast. Breath samples will be collected at baseline (fasting) and at 10, 20, 30, 40, 50, 60 min at first hour of the test, and then at 20, 40 and 60 min during second hour of the test. The samples were analyzed with an IRIS 13C-Analyser System (Wagner, Bremen, Germany).

**Molecular biology methods.** Gene expression of cytokines TNF-a and IL-6 **RNA isolation.** PBMC will be isolated from whole blood by Ficoll density gradient centrifugation. RNA isolation from peripheral blood mononuclear cells (PBMC) will be performed by using TRI Reagent and then, purified on the columns. The amount of total mRNA will be determined by optical density. **Real time PCR**: Expression of TNF-a and IL-6 in *PBMC* will be done by using Real-time PCR method, with the use of QuantiTect TM SYBR1 Green PCR kit (Qiagen, UK) and the Light Cycler480, Roche.

**Genotyping.** DNA will be isolated from peripheral blood leukocytes with the use of commercial kits for genomic DNA isolation. To assess PPAR-y, FTO, IL-6 genotypes, polymerase chain reaction to amplify the regions of the PPAR-y, FTO, IL-6 genes that contain the Pro12Ala, rs9939609 substitutions respectively, will be used. Efficiency of amplification will be confirmed with the use of agarose gel electrophoresis. Then PCR products will be digested with restriction enzymes, and genotypes will be determined after agarose gel electrophoresis. If there is no restriction enzyme recognition site, genotyping will be performed by DNA sequencing. Both RNA isolation and real time PCR will be performed by the Laboratory of Molecular Metabolism at the Faculty of Food Sciences and Nutrition of the Poznań University of Life Sciences (PULS) (<http://www1.up.poznan.pl/mml/>)

**Safety Considerations.** Procedures, laboratory tests, or other measures from baseline to the end of the trial will be checked for safety for patients enrolled to the study. A data monitoring committee from independent institution (Medical University of Poznan, experts of Poznan Medical Ethics Committee for the ethical treatment) will supervise the running of the trial.

**Follow-Up.** The study participants will be assigned either to the CED or the MED diet for 16 weeks. A risk occurring as a result of participation in a research study is minimal. The probability and magnitude of harm (physical, psychological, social, or economic) or discomfort anticipated in the proposed research are not greater, in and of themselves, than those ordinarily encountered in daily lives of the general population or during the performance of routine physical or psychological examinations or tests. Adverse events expected because of the underlying condition, could be connected to allergy or intolerance of served food. To minimizing this risk, potential participants will be interviewed about their adverse experiences with foods during recruitment process. There are not known side effects of the proposed dietary intervention.

**Data Management and Statistical Analysis.**

Data will be collected and analyzed as the project progresses to assure the appropriateness of the research, its design, and subject protections. A data monitoring committee including experts from The Karol Marcinkowski Medical University of Poznan will supervise the running of the trial to ensure investigators are following the protocol, complying with regulatory and Good Clinical Practice (GCP) standards, and collecting and reporting quality data.

Coding: Subjects who will be screened for the study will receive an enrolment code. The order of receiving the test diets will be in accordance with the code of randomization. Once allocated, the volunteer randomization numbers will be used to identify the volunteer during the remainder of the study. A volunteer who, for whatever reason, withdraws or is withdrawn from the study after having been allocated a subject randomization number, will be classified as a dropout.

The G*Power Version 3.1.9.2 software (Universitat Kiel, Germany) was used to calculate a sample size. Power calculations (α = 0.05, power = 0.8) were performed based on expected clinically meaningful between groups differences in weight loss of 4.3 kg, equivalent to 5% weight loss for an 86 kg person. The targeted final sample size per group was determined on at least 70 participants. Statistical analysis will be performed by Statstica software (StatSoft, Inc.). Variables will be checked for normal distribution; nonnormal variables will be log-transformed prior to analysis. For ease of interpretation, arithmetic means and back-transformed variables are presented. Data will be expressed as means at the 95% confidence interval, unless otherwise will be stated. Student t-tests to compare the within-group and between-group changes in the selected outcomes, from baseline to follow up, or from baseline to the value averaged across the four postrandomization time points will be used. The chi-square independence test will be used for categorical variables. Pearson correlation will be used to calculate the correlation coefficient.

**Quality Assurance.** Data will be collected and analyzed as the project progresses to assure the appropriateness of the research, its design, and subject protections. A data monitoring committee including experts from The Karol Marcinkowski Medical University of Poznan will supervise the running of the trial to ensure investigators are following the protocol, complying with regulatory and Good Clinical Practice (GCP) standards, and collecting and reporting quality data.

**Expected Outcomes of the Study.** We expected that the results of this research will serve as a useful tool for the development of dietary advice for abdominally obese individuals (especially postmenopausal women) who prefer native foods items, as a way to improve the anthropometrical and biochemical abnormalities associated with metabolic syndrome. It may be easier for Polish patients to improve their diet with the use of well-known, healthy foods items that are targeted at them, rather than adopting a less familiar dietary pattern, such as the MED. In addition, on the molecular level - it will be possible to explain of the interaction between dietary factors and the genetic predisposition to develop of metabolic syndrome risk factors. The proposed research program is aimed at improving the well-being of Poles and of people living in Central European countries. To achieve the potential benefits associated with the concept of a modern nutritional strategy, we will create an interdisciplinary research team of professionals in field of nutrition, medicine and molecular biology. Additionally, if the therapy is successful, native food items with high nutritive characteristic, for example rapeseed oil, rye bread will be promoted.

**Dissemination of Results and Publication Policy.** The proposed research will be disseminated as publications in an international journals, and also as reports at national and international conferences.

**Duration of the Project.** Dietary intervention will be lasting 16 weeks. Changes in body weight, body composition, waist circumferences, will be measured in four-week intervals (week 0, 4, 8, 12 and 16). Additionally, dietary intake and physical activity level will be checked. Before (week 0) and after dietary intervention period (week 16) changes in visceral fat, biomedical parameters will be done: in systolic and diastolic blood pressure, in risk factors for metabolic syndrome (HDL cholesterol, insulin, triglycerides and glucose), and in inflammatory markers: hcy, lep, hsCRP, TNF-α, IL-6 and adiponectin. In the same time influence of CED and MED on liver function, resting metabolic rate, respiratory quotient and gene expression of inflammatory cytokines: TNF-α, and IL-6, in PBMC, attitude toward food intake will be done. To check associations between genes polymorphism and effectiveness of the dietary intervention and metabolic variables, the centrally obese postmenopausal women will be genotyped for FTO, PPAR-γ,IL-6. A year after the completion of the dietary intervention (week 52) body weight change and changes in risk factors for metabolic syndrome as well as in physical activity, , attitudes toward food intake will be done (Table 1).

Table 1. Intervention scheme

|  | **Screening** | **Baseline evaluation** | **Dietary intervention** | | | | **1-year after end of dietary intervention** |
| --- | --- | --- | --- | --- | --- | --- | --- |
|  | **-2** | **0** | **wk4** | **wk8** | **wk12** | **wk16** | **wkimpro52** |
| **Demographic questionnaires, physician interview** | **x** |  |  |  |  |  |  |
| **FFQ, 3-days dietary diary** |  | **x** | **x** | **x** | **x** | **x** | **x** |
| **Anthropometry (BW, WC, FM, FFM)** |  | **x** | **x** | **x** | **x** | **x** | **x** |
| **Physical activity (IPAQsv)** |  | **x** | **x** | **x** | **x** | **x** | **x** |
| **Blood pressure** |  | **x** |  |  |  | **x** | **x** |
| **DXA (bone mineral density and body composition )** |  | **x** |  |  |  | **x** | **x** |
| **Blood (GLU, INS, TC, LDL-C, HDL-C,TG)** |  | **x** |  |  |  | **x** | **x** |
| **Blood (ins, hcy, adpq, TNFa, IL-6, lep, hsCRP)** |  | **x** |  |  |  | **x** |  |
| **REE, npRQ** |  | **x** |  | **x** |  | **x** | **x** |
| **13C Methacetine breath test** |  | **x** |  |  |  | **x** |  |
| **Blood (TNFa/IL-6 expression)** |  | **x** |  |  |  | **x** |  |
| **Blood (polymorphisms FTO, PPARG, IL-6)** |  | **x** |  |  |  |  |  |
| **Eating inventory (restraint, hunger, disinhibition)** |  | **x** |  |  |  | **x** | **x** |

**Problems Anticipated.** Two of the major concerns to dietary intervention study are the recruitment and retention of eligible study participants for the duration of the study. While recruitment targets seems to be met in the planned study (due to high prevalence of obesity among polish postmenopausal women), retention could be poor. It is widely noted that drop-outs in intervention trials can affect the statistical power of the study. Drop-outs are due to family or work problems, logistic difficulties such as travel and associated costs are frequently cited reason of participants attrition. The next are a lack of early success in weight loss, depression and low self-esteem

**Project Management**

**The PI/Co-PI are responsible for:**

• designing, conducting, and monitoring the protocol

• ensuring that the subjects’ rights, safety, and welfare are protected

• overseeing the informed consent process

• supervising and educating staff involved in the clinical trial

• providing records for inspection

• complying with record-keeping

• overseeing the integrity and analysis of research data

• ensuring that protocols are followed and that data are collected promptly and accurately

• ensuring necessary approvals are obtained

**Trial statistician is responsible for:**

• designing the trial,

• making midway corrections to the conduct of the trial,

• analyzing the data

**Data manager is responsible for:**

• recruiting, screening, and scheduling procedures

• collecting source documents

• providing periodic reports

• applying quality control measures during each stage of data handling to ensure that all data are reliable and have been processed correctly

**Technician staffs are responsible for:**

• blood collection, RNA and DNA isolation, performance of anthropometric, physical activity, nutritional habits assessment

**Ethics.** The final study protocol, including the final version of the Subject Information and Consent Forms, must be approved in writing by an Independent Ethics Committee (The Karol Marcinkowski Medical University of Poznan Bioethics Commission) before enrolment of any subject into the study. Privacy, rights, and welfare of the study subjects will be protected.

Patients will be informed about the study procedures and benefits and risks before obtaining informed consent. Selection process will be explained to prospective subjects in language they can understand. All participants will be provided with researchers’ contact information for questions or comments. Written informed consent form include: project title, description, researchers’ contact information, research procedures, perceived benefits/risks, and any additional information that might be relevant in understanding the research study should be provided for interested individuals to review if they are considering participating and to sign if they would like to participate. One copy will be signed and returned to the researcher and an additional copy should be given to individuals to keep. Care and protection for research participants during the study will be ensured by frequent monitoring and the presence of trained personnel who can respond to emergencies. Data will be collected from standard-of-care procedures to avoid unnecessary risk, particularly for invasive procedures.

**Assessment of risks and benefits:** A risk occurring as a result of participation in a research study is minimal. The probability and magnitude of harm (physical, psychological, social, or economic) or discomfort anticipated in the proposed research are not greater, in and of themselves, than those ordinarily encountered in daily lives of the general population or during the performance of routine physical or psychological examinations or tests. Adverse events expected because of the underlying condition, could be connected to allergy to or intolerance of served food. To minimizing this risk, potential participants will be interviewed about their adverse experiences with foods during recruitment process. There are not known side effects of the intervention.

**Reference****s**

1. Lizcano, F. & Guzmán, G. Estrogen deficiency and the origin of obesity during menopause. *Biomed Res Int*. **2014**, 757461 (2014).
2. Stachowiak, G., Pertyński, T. & Pertyńska-Marczewska, M. Metabolic disorders in menopause. *Prz Menopauzalny* **14**, 59–64 (2015).
3. Deibert, P. *et al.* Effect of a weight loss intervention on anthropometric measures and metabolic risk factors in pre- versus postmenopausal women. *Nutr J.* **6**, 31, 10.1186/1475-2891-6-31 (2007).
4. Aude, Y.W *et al.* The national cholesterol education program diet vs a diet lower in carbohydrates and higher in protein and monounsaturated fat: a randomized trial. *Arch Intern Med*. **164**, 2141-2146 (2004).
5. Eilat-Adar, S., Sinai, T., Yosefy, C. & Henkin, Y. Nutritional recommendations for cardiovascular disease prevention. *Nutrients* **5**, 3646-3683 (2013).
6. Bach-Faig, A. *et al.* Mediterranean Diet Foundation Expert Group. Mediterranean diet pyramid today. Science and cultural updates. *Public Health Nutr.* **14,** 2274-2284 (2011).
7. Davis, C., Bryan, J., Hodgson, J. & Murphy, K. Definition of the Mediterranean Diet; a Literature Review. *Nutrients* **7**, 9139-9153 (2015)
8. Rodriguez-Cano, A. *et al*. Dietary changes associated with improvement of metabolic syndrome components in postmenopausal women receiving two different nutrition interventions. *Menopause* **22**, 758-764 (2015).
9. Babio, N. *et al.* Mediterranean diets and metabolic syndrome status in the PREDIMED randomized trial. *CMAJ.* **186**, E649-E657 (2014).
10. Salas-Salvadó, J. *et al.* Effect of a Mediterranean diet supplemented with nuts on metabolic syndrome status: one-year results of the PREDIMED randomized trial. *Arch Intern Med.* **168**, 2449-2458 (2008).
11. Babio, N. *et al.* Adherence to the Mediterranean diet and risk of metabolic syndrome and its components. *Nutr Metab Cardiovasc Dis.* **19**, 563-570 (2009).
12. Esposito, K., Kastorini, C. M., Panagiotakos, D. B. & Giugliano, D. Mediterranean diet and weight loss: meta-analysis of randomized controlled trials. *Metab Syndr Relat Disord*. **9**, 1–12 (2011).
13. Papadaki, A. & Scott, J. A. The impact on eating habits of temporary translocation from a Mediterranean to a Northern European environment. *Eur J Clin Nutr*. **56**, 455-461 (2002).
14. Olsen, A. et al. Healthy Aspects of the Nordic Diet Are Related to Lower Total Mortality. J Nutr. 141, 639-644 (2011).
15. Alberti, K. G., Zimmet, P., Shaw, J. & IDF Epidemiology Task Force Consensus Group. The metabolic syndrome new worldwide defi nition. Lancet 366, 1059-1062 (2005).
16. Alhassan, S., Kim, S., Bersamin, A., King, A. C. & Gardner, C. D. Dietary adherence and weight loss success among overweight women: results from the A TO Z weight loss study. Int J Obes (Lond). 32, 985-991 (2008).
17. Whitworth, J. A. & Chalmers, J. World health organisation-international society of hypertension (WHO/ISH) hypertension guidelines. Clin Exp Hypertens. 26, 747-752 (2004).
18. Friedewald, W. T., Levy, R. I. & Fredrickson, D. S. Estimation of the concentration of low-density lipoprotein cholesterol in plasma, without use of the preparative ultracentrifuge. Clin Chem. 18, 499-502 (1972).
19. Matthews, D. R. et al. Homeostasis model assessment: insulin resistance and B-cell function from fasting plasma glucose and insulin concentrations in man. Diabetologia 28, 412–419 (1985).
20. Chmurzynska, A., Malinowska, A. M., Twardowska-Rajewska, J. & Gawecki, J. Elderly women: homocysteine reduction by short-term folic acid supplementation resulting in increased glucose concentrations and affecting lipid metabolism (C677T MTHFR polymorphism). Nutrition 29, 841-844 (2013).

**Informed Consent Forms.**

I, the undersigned, confirm that (please tick box as appropriate):

| 1. | I have read and understood the information about the project, as provided in the Information Sheet dated ________________. | 🞏 |
| --- | --- | --- |
| 2. | I have been given the opportunity to ask questions about the project and my participation. | 🞏 |
| 3. | I voluntarily agree to participate in the project. | 🞏 |
| 4. | I understand I can withdraw at any time without giving reasons and that I will not be penalised for withdrawing nor will I be questioned on why I have withdrawn. | 🞏 |
| 5. | The procedures regarding confidentiality have been clearly explained (e.g. use of names, pseudonyms, anonymisation of data, etc.) to me. | 🞏 |
| 6. | The use of the data in research, publications, sharing and archiving has been explained to me. | 🞏 |
| 7. | I have been informed that I will not receive any material benefits from participation in the project, and my participation in this project will only bring health benefits | 🞏 |
| 8. | I certify that I have received a copy of this form with a signature and date. | 🞏 |

**Participant:**

________________________ ___________________________ ________________

Name of Participant Signature Date

**Researcher:**

________________________ ___________________________ ________________

Name of Researcher Signature Date

**ŚWIADOMA ZGODA PACJENTA (Polish)**

Ja niżej podpisany potwierdzam, że: (proszę zaznaczyć odpowiednie pole):

| 1. | Przeczytałem i zrozumiałem dane dotyczące projektu zamieszczone w Informacji dla pacjenta z dnia ________________. | 🞏 |
| --- | --- | --- |
| 2. | Miałam możliwość zadawania pytań dotyczących wszystkich aspektów badań. | 🞏 |
| 3. | Zgadzam się na dobrowolny udział w badaniach | 🞏 |
| 4. | Jestem świadoma faktu, iż w każdej chwili mogę wycofać zgodę na udział w badaniach bez podania przyczyny | 🞏 |
| 5. | Wiem, że moje dane osobowe nigdy nie zostaną ujawnione, a zebrane informacje pozostaną poufne. | 🞏 |
| 6. | Zostałem poinformowany o możliwości wykorzystania zebranych podczas badań danych pomiarowych np. w raportach, publikacjach naukowych | 🞏 |
| 7. | Zostałem poinformowany, że nie otrzymam żadnych korzyści materialnych z tytułu uczestnictwa w w/w badaniach, a mój udział w nich przyniesie wyłącznie korzyści zdrowotne | 🞏 |
| 8. | Poświadczam, że otrzymałam kopię niniejszego formularza opatrzoną podpisem i datą. | 🞏 |

**Uczestnik badania:**

________________________ ___________________________ ________________

Imię i Nazwisko Podpis Data

**Badacz:**

__________________ ___________________________ ________________

Imię i Nazwisko Podpis Data

**Part 2.**

**Budget. Total p**lanned expenses: 592 220 PLN/141 000 €

- **Salary:** 100 000 PLN/23 810 €
- **Overhead**: 122 820 PLN/29 240 €
- **Other cost justification**: 369 400 PLN/87 950 €:

1. Prepare and deliver to each of participant one main meal (dinner).

2. Assessment of the biochemical parameters. Purchase of specific kits, tips and other small the laboratory equipment

3. Purchase of 13C-labelled-methacetin-breath test to assess liver metabolism (necessary for the implementation of the project)

4. Cost of participation in local and international scientific conferences,

5. Purchase of reagents and disposable materials for genetic analysis

6. Purchase of DNA isolation kit, a kit for purification of PCR products,

7. Purchase of office materials

**Other support for the Project**: not applicable

**Collaboration with other scientists or research institutions**

Collaborations with: the Latvia State Institute of Fruit Growing, the Institute of Food Technology of Plant Origin (Poznan University of Life Sciences),

Links to other projects: <https://projekty.ncn.gov.pl/index.php?s=492>, <http://www1.up.poznan.pl/mml/projects.html>

**Curriculum Vitae of investigators**

**1. Joanna Bajerska, PhD**

**Education:** PhD with habilitation (Human Nutrition, 2015), PhD (Human Nutrition, 2006), MSc (Human Nutrition, 2000)

**Current position:** Associate Professor, Institute of Human Nutrition and Dietetics, Dietetic Division, Poznan University of Life Sciences, Poznan, Wojska Polskiego 31, 60-637 Poland. Email: [joanna.bajerska@up.poznan.pl](mailto:joanna.bajerska@up.poznan.pl) Phone +48 61 846 60 56

**Research stay**: six-week professional internship in the Latvia State Institute of Fruit Growing

**Ongoing research projects funding (PI/co-PI/co-applicant)**

1. **Project ID 2013/09/B/NZ9/02365***: Central European balanced diet in metabolic syndrome therapy - a modern nutrition approach (CED-MED) Sources of funding: NCN (The National Science Centre).* 12.03.20014 – 11.03.2018. *Role: PI (50%)*
2. **Project ID 2015/19/B/NZ9/01038:** *Regulation of the level of neuroactive beta-carbolines in the diet based on coffee substitutes. Sources of funding: NCN.* 2016-06-14*–* 13.06.2019*. Role: Co-PI (20%)*

**Research projects completed**

1. **EU-JPI Healthy Diet for a Healthy Life:** *DEDIPAC Knowledge Hu. Dec 2013-Nov 2016. Role:* co-applicant.
2. **Project ID: N N312 124139:** *Evaluation of the possibility to use cranberry-based foods and their waste products in the prevention and treatment of periodontal disease*. Sources of funding: NCN. *Nov 2010-May 2015. Role: co-PI*.

**Recent publications**

1. Józefczuk J, Malikowska K, Glapa A, Stawińska-Witoszyńska B, Nowak JK, **Bajerska J**, Lisowska A, Walkowiak J. (2017) Mulberry leaf extract decreases digestion and absorption of starch in healthy subjects-A randomized, placebo-controlled, crossover study. Adv Med Sci. 2017 Sep;62(2):302-306.
2. Mildner-Szkudlarz S, **Bajerska J,** Górnaś P, et al. (2016) Physical and Bioactive Properties of Muffins Enriched with Raspberry and Cranberry Pomace Powder: A Promising Application of Fruit By-Products Rich in Biocompounds. *Plant Foods Hum Nutr.* 2016 Apr
3. **Bajerska J**. Mildner-Szkudlarz S. Górnaś P., and D. Seglina (2016) The effects of muffins enriched with sour cherry pomace on glycemic response, energy intake, satiety, and acceptability: A randomized crossover trial. *J Sci Food Agric.* 96(7): 2486-93.
4. Lochocka K., **Bajerska J**., Glapa A., et al. (2015) Green tea extract decreases starch digestion and absorption from a test meal in humans: a randomized, placebo-controlled crossover study. *Sci Rep.* 2015 Jul 30;5:12015.
5. Mildner-Szkudlarz S, Siger A, Szwengiel A, and **Bajerska J.** (2015) Natural compounds from grape by-products enhance nutritive value and reduce formation of CML in model muffins. *Food Chem.* 2015 1;172:78-85.
6. **Bajerska J**, Mildner-Szkudlarz S, Walkowiak J. (2015) Effects of Rye Bread Enriched with Green Tea Extract on Weight Maintenance and the Characteristics of Metabolic Syndrome Following Weight Loss: A Pilot Study. *J Med Food.* 18(6):698-705.
7. **Bajerska J,** Chmurzynska A, Mildner-Szkudlarz S, and Drzymała-Czyż S. (2015) Effect of rye bread enriched with tomato pomace on fat absorption and lipid metabolism in rats fed a high-fat diet *J Sci Food Agric.* 95(9):1918-24
8. **Bajerska J,** Woźniewicz M, Suwalska A, and Jeszka J. (2014) Eating patterns are associated with cognitive function in the elderly at risk of metabolic syndrome from rural areas. *Eur Rev Med Pharmacol Sci.* 18(21):3234-45.

**2. Agata Chmurzynska, PhD**

**Education:** 2012: habilitation in human nutrition, Warsaw University of Life Sciences

2006: Ph.D. in biology (genetics), Adam Mickiewicz University, Poznań

2002: M.Sc. in biotechnology (molecular diagnostics), Agricultural University of Poznań

**Current position:** Associate professor, Institute of Human Nutrition and Dietetics, Poznan University of Life Sciences, Poznan, Wojska Polskiego 31, 60-637 Poland. Email: [joanna.bajerska@up.poznan.pl](mailto:joanna.bajerska@up.poznan.pl) Phone +48 61 846 6056

Director of the Institute of Human Nutrition and Dietetics, the Human Nutrition and Nutrigenomics Division, Poznan University of Life Sciences, Founder and the director of the Molecular Metabolism Laboratory,

**Research stay**: May 2011-October 2011; BioInfoBankInstitute, Poland; fellowship; Implementation of the bioinformatic system for next generation sequencing data assembly.

January 2014-February 2014; Monell Chemical Senses Center, Philadelphia, USA; visiting scientist (Dr. Danielle Reed's lab)

April 2015-June 2015; Nutrition Research Institute, University of North Carolina at Chapel Hill, USA; visiting professor (Zeisel's Lab)

**Research Projects**

1. Assessment of choline intake and choline metabolism in children (The Regional Operational Program for Mazowieckie Voivodeship; PI: Agata Chmurzynska, PhD). Collaboration with the Institute of Food Technology of Plant Origin (Poznan University of Life Sciences), the Children's Memorial Health Institute (Warsaw) and Nutropharma company.
2. The effect of diet of pregnant rats on inheritance of DNA methylation patterns. (NCN, Co-PI. Collaboration with the Department of Genetics and Animal Breeding, Poznan University of Life Sciences
3. Relationships between fat discrimination, frequency of eating high-fat foods, and gene polymorphism - determination of body weight and lipid metabolism. NCN, PI
4. The study of the influence of intestinal and cellular iron and folate transporters on bioavailability of these micronutrients in the organism (NCN, Co-PI)

**Recent publications**

1. Nowacka-Woszuk J, Madeja ZE, **Chmurzynska A.** Prenatal caloric restriction alters lipid metabolism but not hepatic Fasn gene expression and methylation profiles in rats. BMC Genet. 2017 Aug 15;18(1):78. doi: 10.1186/s12863-017-0544-0.
2. **Chmurzynska A,** Malinowska AM, Twardowska-Rajewska J, Gawecki J. Rs6586282 of the CBS Gene: Its Lack of Effect on Homocysteine Concentrations, and Interaction Effects on Body Weight in Elderly Women. Int J Vitam Nutr Res. 2017 Aug 15:1-7. doi: 10.1024/0300-9831/a000284.
3. Ramos-Lopez O, Milagro FI, Allayee H, **Chmurzynska A,** et al. Guide for Current Nutrigenetic, Nutrigenomic, and Nutriepigenetic Approaches for Precision Nutrition Involving the Prevention and Management of Chronic Diseases Associated with Obesity. J Nutrigenet Nutrigenomics. 2017;10(1-2):43-62. doi: 10.1159/000477729. Epub 2017 Jul 8.
4. Mikołajczyk-Stecyna J, Malinowska AM, **Chmurzynska A.** TAS2R38 and CA6 genetic polymorphisms, frequency of bitter food intake, and blood biomarkers among elderly woman. Appetite. 2017 Sep 1;116:57-64. doi: 10.1016/j.appet.2017.04.029. Epub 2017 Apr 25.
5. **Chmurzynska A,** Mlodzik MA. [Genetics of fat intake in the determination of body mass.](https://www.ncbi.nlm.nih.gov/pubmed/28294082) Nutr Res Rev. 2017 Jun;30(1):106-117. doi: 10.1017/S0954422417000014. Epub 2017 Mar 15.
6. **Chmurzynska A,** Malinowska AM, Twardowska-Rajewska J, Gawecki J. Elderly women: homocysteine reduction by short-term folic acid supplementation resulting in increased glucose concentrations and affecting lipid metabolism (C677T MTHFR polymorphism). Nutrition. 2013 Jun;29(6):841-4. doi: 10.1016/j.nut.2012.09.015. Epub 2013 Jan 5.

**3. Prof. Jarosław Walkowiak, PhD, MD**

**Education:** 1991 - MD, Poznań University School of Medicine (PUMS), Faculty of Medicine, Poland (cum lauda)

1995 - PhD, PUMS, Poland

2007 - Associate Professor (habilitation) PUMS, Poland

2007 - Professor (title), PUMS, Poland

**Current position:** From 2012 - Head of Department of Pediatric Gastroenterology and Metabolic Diseases, PUMS

From 2012 - Vice-rector for Science & Foreign Relations, PUMS

**Research stay**: 1989 clinical training (IFMSA exchange), University of Erlangen, Germany, 1 month

1991 scientific training (EISEC exchange) University of Essen, Germany, 1 month

2000 scientific and clinical training (University exchange), University of Kiel, Germany (1 month)

2000 scientific fellowship (Stephan Batory Foundation), University of Kiel, Germany (4 months)

2001- 2002 scientific fellowship (Nutricia Foundation), University of Kiel, Germany & University of Kuopio, Finland (12 months)

**Research Projects**

DNA methylation as a factor modifying the response to thiopurine in patients with non-specific inflammatory bowel disease – NCN, PI:

Metabolic consequences of CLA supplementation in overweight and obese subjects. Fundacja Nutricia Foundation research grant, PI.

Central European balanced diet in metabolic syndrome therapy - a modern nutrition approach (CED-MED) Sources of funding: NCN, Co-PI

The risk of atherosclerosis in cystic fibrosis patients influence and its relation to exogenous and endogenous expression of the disease: NCN, Co-PI

**Recent publications**

1. Krzyżanowska P, Drzymala-Czyż S, Rohovyk N, Bober L, Moczkco J, Rachel M, Walkowiak J. Prevalence of vitamin K deficiency and associated factors in non-supplemented cystic fibrosis patients. Arch Argent Pediatr. 2018 Feb 1;116(1):e19-e25. doi: 10.5546/aap.2018.eng.e19. English, Spanish.
2. Mytyk A, Lazowska-Przeorek I, Karolewska-Bochenek K, Kakol D, Banasiuk M, Walkowiak J, Albrecht P, Banaszkiewicz A. Clear-Liquid Versus Low-Fibre Diet in Bowel Cleansing for Colonoscopy in Children: A Randomized Trial. J Pediatr Gastroenterol Nutr. 2017 Nov 3. doi: 10.1097/MPG.0000000000001832. [Epub ahead of print]
3. Walkowiak J, Malikowska K, Glapa A, Bogdański P, Fidler-Witoń E, Szulińska M, Chudzicka-Strugała I, Miśkiewicz-Chotnicka A, Mądry E, Lisowska A. Conjugated linoleic acid does not affect digestion and absorption of fat and starch-a randomized, double-blinded, placebo-controlled parallel study. J Breath Res. 2017 Dec 6;12(1):016010. doi: 10.1088/1752-7163/aa872d.
4. Drzymała-Czyż S, Szczepanik M, Krzyżanowska P, Duś-Żuchowska M, Pogorzelski A, Sapiejka E, Juszczak P, Lisowska A, Koletzko B, Walkowiak J. Serum Phospholipid Fatty Acid Composition in Cystic Fibrosis Patients with and without Liver Cirrhosis. Ann Nutr Metab. 2017;71(1-2):91-98. doi: 10.1159/000477913. Epub 2017 Jul 22.
5. Drzymała-Czyż S, Janich S, Klingler M, Demmelmair J, Walkowiak J, Koletzko B. Whole blood glycerophospholipids in dried blood spots - a reliable marker for the fatty acid status. Chem Phys Lipids. 2017 Oct;207(Pt A):1-9. doi: 10.1016/j.chemphyslip.2017.06.003. Epub 2017 Jun 29.
6. Nowak JK, Wojsyk-Banaszak I, Mądry E, Wykrętowicz A, Krzyżanowska P, Drzymała-Czyż S, Nowicka A, Pogorzelski A, Sapiejka E, Skorupa W, Szczepanik M, Lisowska A, Walkowiak J. Increased Soluble VCAM-1 and Normal P-Selectin in Cystic Fibrosis: a Cross-Sectional Study. Lung. 2017 Aug;195(4):445-453. doi: 10.1007/s00408-017-0029-y. Epub 2017 Jun 23.

**Other research activities of the investigators.** A relevant information in Bajerska CV are given

**Financing and Insurance**. Payment of clinical research subjects – not applicable.

**Insurance in clinical research. I**n the event of adverse events suffered by subjects in a clinical trial – insurance policy 436000117102 Ergo Hestia S.A. All participants possess also the general health insurance
